# Salvianolic-Acid-B-Loaded HA Self-Healing Hydrogel Promotes Diabetic Wound Healing through Promotion of Anti-Inflammation and Angiogenesis

**DOI:** 10.3390/ijms24076844

**Published:** 2023-04-06

**Authors:** Guoying Zhou, Jiayan Zhu, Liang Jin, Jing Chen, Ruojiao Xu, Yali Zhao, Tingzi Yan, Haitong Wan

**Affiliations:** 1 College of Life Science, Zhejiang Chinese Medical University, Hangzhou 310053, China; gyzhou87@126.com (G.Z.);; 2 College of Material, Chemistry and Chemical Engineering, Hangzhou Normal University, Hangzhou 311121, China

**Keywords:** diabetic wound healing, self-healing hydrogel, salvianolic acid B, anti-inflammation, angiogenesis

## Abstract

Inflammatory dysfunction and angiogenesis inhibition are two main factors leading to the delayed healing of diabetic wounds. Hydrogels with anti-inflammatory and angiogenesis-promoting effects have been considered as promising wound care materials. Herein, a salvianolic acid B (SAB)-loaded hyaluronic acid (HA) self-healing hydrogel (HA/SAB) with anti-inflammatory and pro-angiogenesis capacities for diabetic wound healing is reported. The HA hydrogel was prepared via the covalent cross-linking of aldehyde groups in oxidized HA (OHA) and hydrazide groups in adipic dihydrazide (ADH)-modified HA (HA-ADH) with the formation of reversible acylhydrazone bonds. The obtained HA hydrogel exhibited multiple favorable properties such as porous structures, excellent self-healing properties, a sustainable release capacity of SAB, as well as excellent cytocompatibility. In addition, the effects of the SAB-loaded HA self-healing hydrogel were investigated via a full-thickness skin defect model using diabetic rats. The HA/SAB hydrogel showed enhanced skin regeneration effects with accelerated wound closure, shorter remaining dermal space length, thicker granulation tissue formation, and more collagen deposition. Furthermore, reduced inflammatory response and enhanced vascularization were found with HA/SAB2.5 hydrogel-treated wounds, indicating that the hydrogel promotes diabetic wound healing through the promotion of anti-inflammation and angiogenesis. Our results suggest that the fabricated SAB-loaded HA self-healing hydrogel is promising as a wound dressing for the treatment of diabetic wounds.

## 1. Introduction

Diabetes mellitus (DM) is accompanied by several complications such as hyperglycemia, peripheral vascular disease, persistent infection, and tissue damage [1]. Hyperglycemia-induced persistent inflammation and insufficient angiogenesis are two main factors leading to the dysregulation of diabetic wound healing [2]. Skin regeneration in normal wounds can be divided into four stages: hemostasis, inflammation, proliferation, and tissue remodeling, among which, the transition from the pro-inflammatory towards the anti-inflammatory phase at the inflammation stage is of great importance for the healing of skin [3]. However, high blood glucose environments impede the pro- towards anti-inflammatory phase switch in diabetic wounds [4]. In addition, the increased production of reactive oxygen species (ROS) results in the upregulation of pro-inflammatory factors, which further prolongs the pro-inflammatory stage [5,6]. As a result, the entire healing process is prolonged in diabetic wounds. On the other hand, hyperglycemia impairs angiogenesis by reducing hypoxia-inducible factor-1 (HIF-1) and inhibiting subsequent vascular endothelial growth factor (VEGF) signaling [7,8]. The reduced angiogenesis results in decreased blood vessel density and an insufficient supply of oxygen and nutrients, ultimately leading to the delayed formation of granulation tissue [9,10]. Consequently, there is an urgent need to develop effective therapeutic approaches with anti-inflammatory and pro-angiogenesis capacities to accelerate diabetic wound healing.

Hydrogels have been recognized as promising candidates for the treatment of wounds due to their excellent biocompatibility, biomimetic structures, and desirable physical properties [11,12]. When applied to wounds, hydrogel wound dressings can absorb excess tissue secretions, protect the wound from infection, and maintain a good moist environment [12]. For example, a food gum hydrogel doped with polydopamine nanoparticles fabricated by Shen et al. exhibited rapid shape adaptability, allowing it to fit to any shape of wound, protect the wound, and provide a moist environment for better healing of the wound [13]. Currently, many studies are focused on the application of polysaccharide-based hydrogels such as hyaluronic acid (HA) as wound dressing materials [14]. As a main component of extracellular matrices, HA can modulate multiple cellular behaviors such as cell adhesion, proliferation, and differentiation [15]. In addition, the functional side groups on the polymer chain of HA can be appropriately modified to achieve different therapeutic effects [16,17]. It has been known that the different molecular weights of HA can affect the physical properties of the prepared hydrogels such as the modification efficiency of the hydrogel precursors, mechanical properties, degradation profile, drug release behaviors, and so on [18]. Previous studies have shown low-molecular-weight HA (10–500 kDa)-based hydrogels exhibited good mechanical properties with storage moduli ranging from dozens to hundreds, which are suitable for application in wounds and proven to exhibit excellent therapeutic effects in wound healing [19,20,21]. Therefore, HA with Mw 200−400 kDa was selected for our studies.

Nevertheless, the traditional natural polymer-based hydrogels can be easily damaged by external tension, resulting in loss of function, possible infection, and inflammation [22]. To solve this problem, self-healing hydrogels generated by dynamic covalent or non-covalent bonds can maintain structural and functional integrity due to their recoverable bonds upon damage [23,24]. In addition, self-healing hydrogels usually exhibit favorable injectability and moldability, which are suitable for applications in irregular wounds [25]. For example, Zhao et al. developed a self-healing HA nanocomposite hydrogel based on aldehyde-modified sodium hyaluronate (AHA), hydrazide-modified sodium hyaluronate (ADA), and aldehyde-modified cellulose nanocrystals (oxi-CNC) [26]. With the sustained release of platelet-rich plasma (PRP), this hydrogel significantly promoted full-thickness skin wound healing. According to the study by Chi et al., thiol-modified poly (γ-glutamic acid) (γ-PGA-SH) and oxidized hyaluronic acid (HA-CHO) formed a self-healing hydrogel by a thiol-aldehyde addition reaction [27]. In a full-thickness skin defect model, this hydrogel exhibited enhanced wound healing compared to the commercial dressing by facilitating angiogenesis and promoting collagen deposition. In another study by Gu et al., adipic-acid-dihydrazide-modified hyaluronic acid (HA-ADH), dopamine-functionalized oxidized hyaluronic acid (OHA-Dop), and aldehyde-terminated Pluronic F127 (AF127) were cross-linked to form a double-network hydrogel with adhesive and self-healing properties [19]. In vivo experimental results showed the potential of this hydrogel to accelerate wound closure and improve skin regeneration.

The porous structure of hydrogels endows them with a drug delivery function. For instance, Shen et al. constructed a polydopamine-incorporated dextran hydrogel, which can serve as a drug delivery vector for the sustained release of antibiotics and possesses great promise as a wound dressing [28]. It has been suggested that hydrogels loaded with anti-inflammatory or angiogenic bioactive components can promote diabetic wound healing [29]. However, most of the drugs delivered are antibiotics and growth factors, which have disadvantages such as bacterial resistance, biological toxicity, and high cost [30]. Traditional Chinese medicine or their bioactive components, with versatility, hypotoxicity, and cost-effective advantages, can be loaded in hydrogel, offering a new possibility for diabetic wound healing [31]. For example, Qian et al. developed a supramolecular hydrogel based on hydroxypropyl chitosan (HPCS) and poly (N-isopropylacrylamide) (PNIPAM), with the loading of dipotassium glycyrrhizinate (DG) as the antimicrobial and anti-inflammatory drug for wound repair [32]. In addition, a chitosan-based hydrogel loaded with a flavonoid fraction has been developed and was shown to be promising as a diabetic wound dressing through its antioxidant properties [33]. Furthermore, Guo et al. reported the preparation of a paeoniflorin-loaded hyaluronic acid hydrogel (HA-PF), which can achieve the transition of macrophages from the pro-inflammatory M1 to anti-inflammatory M2 phenotype, resulting in the significant promotion of diabetic wound healing [34].

Salvianolic acid B (SAB) is a major water-soluble component of the traditional Chinese medicine, *Salvia miltiorrhiza*, with extensive pharmacological activities including anti-inflammatory, antioxidant, and pro-angiogenesis [35]. According to the study by Sun et al., SAB significantly decreased the mRNA expression level of IL-1β, IL-6, and TNF-α, and downregulated the protein expression of TLR4, p-p65, and p-IκBα in LPS-stimulated THP-1-macrophages [36]. Liu et al. reported that SAB can eliminate and inhibit ROS generation through the regulation of the Nrf2/Keap1 pathway [37]. In addition, it was shown to have enhanced cerebral angiogenesis and increased microvessel density after SAB treatment in a mice distal middle cerebral artery occlusion (dMCAO) model [38].

In this study, we designed an SAB-loaded HA self-healing hydrogel to accelerate diabetic wound healing, benefiting from the anti-inflammatory and pro-angiogenesis capacities of SAB. The microstructures, rheological property, degradation performance, and drug release profile of the hydrogels were studied. Furthermore, the efficacy of the HA/SAB hydrogel in promoting diabetic wound healing was investigated via a full-thickness skin defect model using diabetic SD rats. The results are reported herein.

## 2. Results

### 2.1. Preparation and Characterization of the Hydrogels

**Figure 1:**
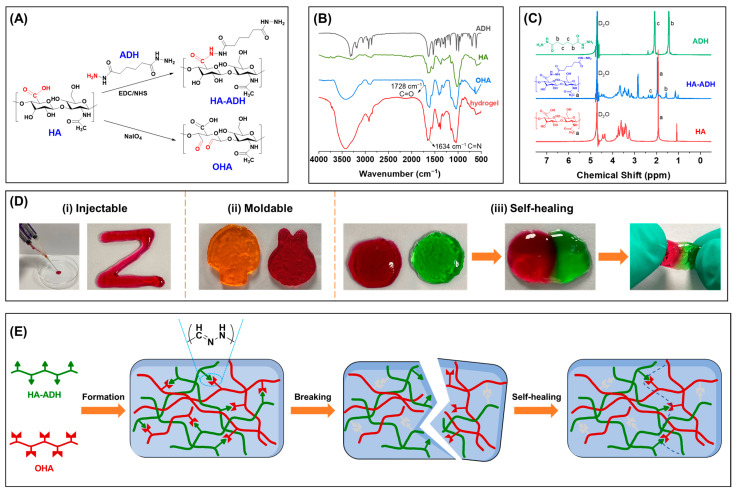
Characterization of the basic properties of the HA hydrogel. (**A**) Synthesis of adipic dihydrazide (ADH)-modified HA (HA-ADH) and oxidized HA (OHA). (**B**) FT-IR spectra of HA, ADH, OHA, and HA/SAB2.5 hydrogel. (**C**) ^1^H NMR spectrum of HA, ADH, and HA-ADH. (**D**) Photographs of the injectable (**i**), moldable (**ii**), and self-healing (**iii**) properties of the HA hydrogel. (**E**) Schematic illustration of the HA hydrogel formation and self-healing properties.

The schematic illustration for the fabrication of OHA and HA-ADH is shown in Figure 1A. The FT-IR spectra of the HA, ADH, OHA, and HA/SAB2.5 hydrogel are shown in Figure 1B. A new absorption peak at 1728 cm^−1^ appears in the spectrum of OHA compared to that of HA. This can be attributed to the aldehyde groups oxidized from the adjacent sugar hydroxyl groups, indicating the successful synthesis of OHA. Additionally, the peak at 1728 cm^−1^ for aldehyde groups in OHA nearly disappears after the formation of hydrogel. Furthermore, a new peak corresponding to the stretching of the C=N groups at around 1634 cm^−1^ appears in the hydrogel compared to OHA. Taken together with the decrease in aldehyde groups and the appearance of C=N groups, these results can demonstrate the formation of acylhydrazone bonds in hydrogels through the covalent reaction of aldehyde groups in OHA and hydrazine groups in HA-ADH, confirming the successful formation of hydrogels. Similar results were also found by others [26,39]. The ^1^H NMR spectrum was applied to confirm the synthesis of HA-ADH. As shown in Figure 1C, HA showed a characteristic peak at 1.92 ppm (methyl proton of acetamido moiety, marked as “a”), while ADH showed characteristic peaks at 1.44 ppm and 2.09 ppm (methylene proton, marked as “b” and “c”, respectively). Compared with the ^1^H NMR spectrum of HA and ADH, HA-ADH showed both the methyl characteristic peak (“a”, 1.96 ppm) of HA and the methylene characteristic peaks (“b” and “c”) of ADH. These results confirmed the successful introduction of hydrazide groups into the HA dimer unit.

The injectable, moldable, and self-healing properties of the HA hydrogels have also been evaluated. As shown in Figure 1D(i), the hydrogels exhibited favorable injectability without blocking the needle, which can be applied conveniently to the wound site in situ. Additionally, the shape of the hydrogels could be controlled by the replacement of molds (Figure 1D(ii)). Such rapid shape adaptability could enable the hydrogel to fit to any type of wound. Beside this, the self-healing property was evaluated based on the reconstruction of two pre-gelled circular hydrogels with green and red colors, respectively (Figure 1D(iii)). It was found that the two circular hydrogels could combine into a whole piece, strong enough to lift and extend, indicating an excellent self-healing capacity. This self-healing ability was mainly attributed to the formation of dynamic, reversible acylhydrazone bonds between the aldehyde groups in OHA and the hydrazide groups in HA-ADH between the hydrogel networks. Such self-healing capacities endow hydrogels with a rapid recovery rate upon damage caused by external pressures and provide continuous protection for skin wounds. Figure 1E shows the schematic illustration of the HA hydrogel formation and the mechanism of self-healing properties. The HA hydrogel was formed by reacting HA-ADH and OHA, forming reversible acylhydrazone bonds, and therefore endowing the hydrogels with good self-healing properties. When the hydrogel is destroyed, the broken reversible acylhydrazone bonds can spontaneously integrate to form a new cross-linked network structure, so that the self-healing hydrogel can recover its original structure and physical properties.

**Figure 2:**
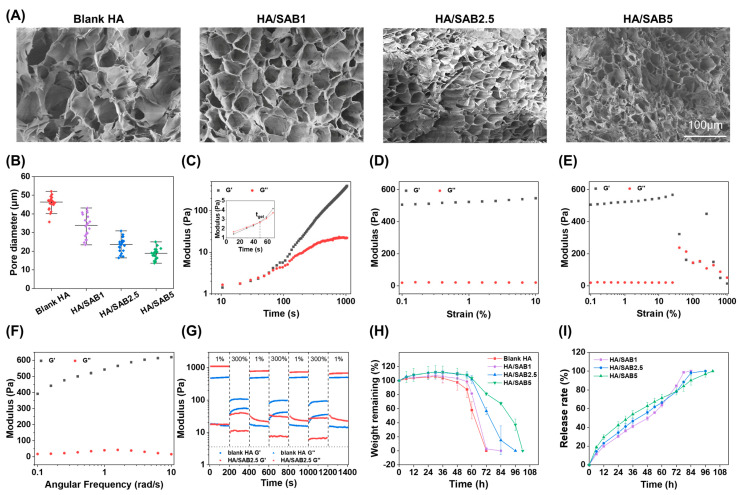
(**A**) SEM images of the blank HA and salvianolic acid B (SAB)-loaded HA hydrogels (HA/SAB1, HA/SAB2.5, and HA/SAB5) (Scale bar = 100 μm). (**B**) Pore size distribution of the blank HA, HA/SAB1, HA/SAB2.5, and HA/SAB5 hydrogels (n = 20). (**C**) Storage modulus (G’) and loss modulus (G’’) of the HA hydrogel cross-linked by adipic dihydrazide (ADH)-modified HA (HA-ADH) and oxidized HA (OHA) at different times at 1% strain and 10 rad/s angular frequency. (**D**,**E**) Strain sweep of the HA hydrogel with strain from 0.1% to 1000% at an angular frequency of 10 rad/s. (**F**) Angular frequency sweep of the HA hydrogel from 0.1 to 10 rad/s at a constant strain of 1%. (**G**) Self-healing property of the blank HA and HA/SAB2.5 hydrogels at continuous step strain test of 1% and 300% strain every 200 s at an angular frequency of 10 rad/s. (**H**) Swelling and degradation behaviors of the blank HA, HA/SAB1, HA/SAB2.5, and HA/SAB5 hydrogels in PBS at 37 °C. (**I**) SAB release profile of the HA/SAB hydrogels in PBS at 37 °C. Data represent mean ± SD, n = 3.

The microstructures of hydrogels were observed by SEM (Figure 2A). Firstly, the internal morphologies of all the hydrogels displayed porous microstructures. Besides that, the blank HA hydrogel showed a larger pore size than all HA/SAB hydrogels. Additionally, the quantified data of pore size is summarized in Figure 2B, and there was found to be a negative correlation between the pore size and the SAB concentration loaded in HA/SAB hydrogels. The quantified values of the pore size of blank HA, HA/SAB1, HA/SAB2.5, and HA/SAB5 were (45.87 ± 3.93 μm), (33.70 ± 6.04 μm), (22.75 ± 4.21 μm), and (18.24 ± 3.04 μm), respectively.

The rheological properties of the hydrogels were investigated by a rheometer. A time sweep test was firstly performed to investigate the gelation time of the hydrogels. As presented in Figure 2C, both the storage modulus (G’) and loss modulus (G”) increased at the beginning, with G’ being smaller than G’’. With the gradual formation of the acylhydrazone bonds between HA-ADH and OHA, G’ and G’’ became comparable to each other at about 48 s, confirming the formation of the gel from the liquid phase. After the hydrogel was completely gelled, a strain sweep test was conducted to study the linear viscoelasticity of the hydrogel. During the strain sweep range from 0.1% to 10% (Figure 2D) and the angular frequency sweep range from 0.1 to 10 rad/s (Figure 2F), the G’ was higher than G’’ without significant change. However, G’ was found to experience a sharp decrease and became smaller than G’’ when subjected to about 63.1% strain, indicating the collapsed state of the hydrogel network with a strain above 63.1%. Furthermore, the self-healing behavior of the blank HA and HA/SAB2.5 hydrogels was investigated by periodic strain changes of 1% and 300% every 200 s, with a frequency of 10 rad/s (Figure 2G). The G’ and G’’ leveled off, with G’ being higher than G’’ at a low strain of 1%. After applying a large strain of 200%, the G’ significantly decreased from 508 to 45.6 Pa and became lower than G’’ (102 Pa), illustrating the damage of the hydrogel network. Once the strain was restored to 1%, both G’ and G’’ instantly returned to the initial value. After experiencing an additional two periodic strain changes, the hydrogel exhibited the same values of G’ and G’’ up to the first cycle, indicating the excellent self-healing property of the HA hydrogel. After the loading of SAB, the HA/SAB2.5 hydrogel exhibited enhanced mechanical properties (with G’ value of 1090 Pa) in contrast to the blank HA hydrogel (with G’ value of 508 Pa). This is because the addition of SAB with abundant phenolic hydroxyl groups can enhance the hydrogen-bond interactions between the SAB-loaded hydrogels, leading to higher cross-linking density and improved mechanical properties. However, the self-healing properties of the HA/SAB2.5 hydrogel were slightly reduced after SAB loading. It was found that the storage modulus (G’) of the HA/SAB2.5 hydrogel could recover to 70% of the original G’ value after three cycles of periodic strain change. The slightly reduced self-healing property in drug-loaded hydrogel might have been due to the formation of hydrogen bonds between phenolic hydroxyl groups in SAB and hydrazide groups in HA-ADH, which restricted the number of remaining hydrazide groups from forming the reversible acylhydrazone bonds, and thus reduced the self-healing abilities. Taken together, the loading of SAB can enhance the mechanical properties of HA hydrogel to prevent rupture, and at the same time, maintain 70% of self-healing abilities to provide rapid structure recovery of hydrogels upon damage.

The degradability of the hydrogels was evaluated by measuring the weight losses of the wet hydrogels. As shown in Figure 2H, all the hydrogels presented similar swelling characteristics, showing that the water uptake content reached the maximum after immersion in PBS for 36 h, and after that, the hydrogels began to degrade. The blank HA group showed the fastest degradation rate, followed by HA/SAB1, HA/SAB2.5, and HA/SAB5.

The release rates of SAB from different HA/SAB hydrogels were evaluated to confirm whether the HA/SAB hydrogels can realize controlled release of SAB. As shown in Figure 2I, the cumulative release of SAB for HA/SAB1, HA/SAB2.5, and HA/SAB5 was 30%, 34%, and 42%, respectively, in the first 24 h. After 48 h of incubation, about 50%, 56%, and 63% of SAB was released from HA/SAB1, HA/SAB2.5, and HA/SAB5, respectively. Notably, all the hydrogels exhibited stable release behavior over time with no obvious initial burst release. These results suggest that the HA/SAB hydrogels might provide a suitable and stable supply of SAB during diabetic wound healing.

### 2.2. Cytocompatibility Evaluation of the Hydrogels

**Figure 3:**
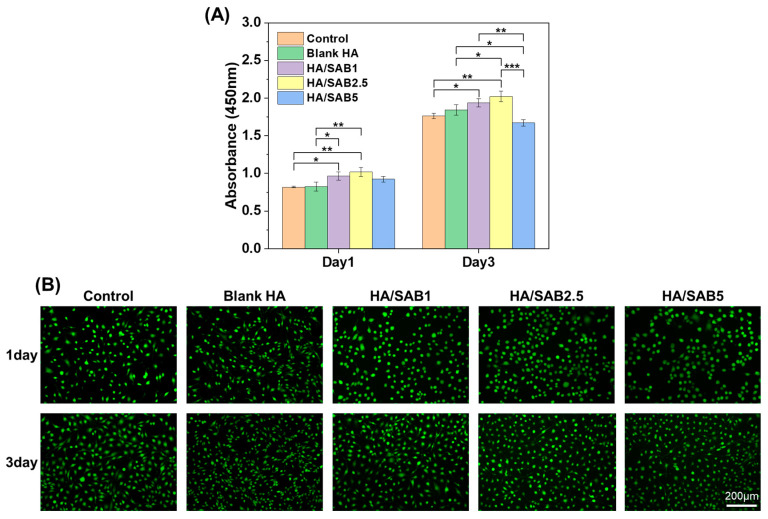
Cytocompatibility of the blank HA and salvianolic acid B (SAB)-loaded HA hydrogels (HA/SAB1, HA/SAB2.5, and HA/SAB5) in vitro. Cytotoxicity analysis of the HA hydrogels with CCK-8 assay (**A**) and LIVE/DEAD staining assay (**B**) using NIH/3T3 cells on day 1 and 3. (Scale bar = 200 μm). Data represent mean ± SD, n = 3; * *p* < 0.05; ** *p* < 0.01; *** *p* < 0.001.

The cytocompatibility of the hydrogels was evaluated by CCK-8 and LIVE/DEAD staining assays. The results of the CCK-8 assay (Figure 3A) indicated that all hydrogels showed no significant cytotoxicity to NIH/3T3 cells within 24 and 72 h. More importantly, the HA/SAB1 and HA/SAB2.5 hydrogels could promote cell proliferation in comparison with the control and blank HA hydrogel groups. In addition, a LIVE/DEAD staining assay was performed for visual verification in Figure 3B, where the apparent green fluorescence indicated the excellent cytocompatibility of blank HA and all HA/SAB hydrogels. Overall, the cellular experiments indicated that the HA hydrogels could promote cell proliferation and exhibit excellent biocompatibility.

### 2.3. Inflammatory Regulation of SAB In Vitro

**Figure 4:**
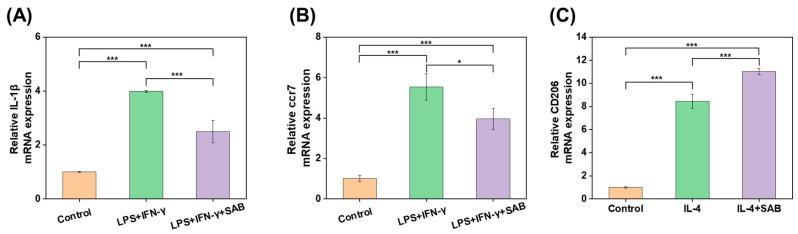
The mRNA expression level of (**A**) IL-1β and (**B**) ccr7 in (LPS + IFN-γ)-stimulated THP-1-derived macrophages following treatment of salvianolic acid B (SAB, 100 μM). (**C**) The mRNA expression level of CD206 in IL-4-stimulated THP-1-derived macrophages following treatment of SAB (100 μM). Data represent mean ± SD, n = 3; * *p* < 0.05; *** *p* < 0.001.

The inflammatory regulation activities of SAB were studied by RT-qPCR. After treatment with LPS and IFN-γ, the mRNA expression level of the pro-inflammatory cytokines IL-1β and ccr7 increased significantly in contrast to the control group, while the overexpression of IL-1β and ccr7 was suppressed significantly by treatment with SAB (Figure 4A,B). On the other hand, IL-4 treatment significantly increased the expression of the M2 marker CD206, indicating the successful stimulation of the M2 phenotype. Additionally, the CD206 expression was elevated further by SAB, suggesting the role of SAB in promoting M2 polarization.

### 2.4. In Vivo Experiment, Histology and Immunofluorescence Staining

**Figure 5:**
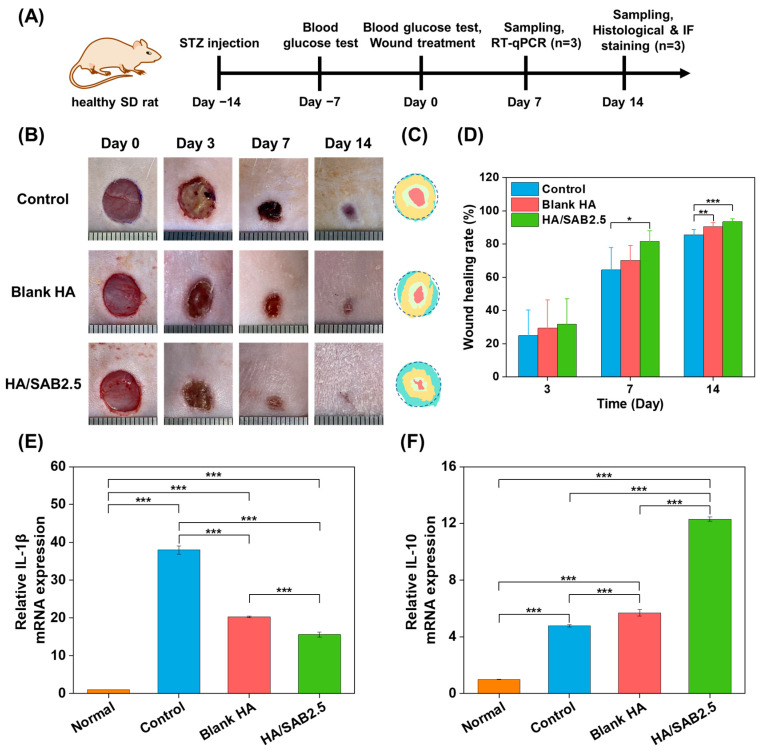
(**A**) Schematic of the establishment, treatment, and assessment of diabetic wound model. (**B**) Representative photographs of diabetic wounds on day 0, 3, 7, and 14 for control, blank HA, and salvianolic acid B (SAB)-loaded HA hydrogel (HA/SAB2.5). (**C**) Schematic diagram of the wound healed by different treatments during 14 days. (**D**) Quantitative data of wound healing rate on day 3, 7, and 14 following treatment of blank HA hydrogel and HA/SAB2.5 hydrogel, n = 6. (**E**,**F**) The mRNA expression level of IL-1β and IL-10 in wound sites on day 7 following treatment of blank HA hydrogel and HA/SAB2.5 hydrogel, n = 3. Data represent mean ± SD; * *p* < 0.05; ** *p* < 0.01; *** *p* < 0.001.

A full-thickness skin defect model was established on the backs of diabetic rats and treated with blank HA and HA/SAB2.5 hydrogels to investigate the properties of the HA hydrogels in promoting wound healing (Figure 5A). Figure 5B shows the representative optical images of gross wound contraction in the different groups on days 0, 3, 7, and 14. It can be seen that the wound sites with initial diameters of 10 mm gradually decreased with time in all three groups, while the HA/SAB2.5 hydrogel group showed the fastest wound healing rate. Figure 5C shows a clear change in wound size in different groups at days 0, 3, 7, and 14. The quantitative values of the wound healing rate over different days are shown in Figure 5D. On day 3, there was no significant difference between the control and hydrogels. On day 7, the HA/SAB2.5-treated group showed a much faster wound healing rate (81.62 ± 6.43%) compared to the control (64.44 ± 13.54%) and blank HA-treated (70.18 ± 8.97%) groups. In order to investigate the effects of blank HA and HA/SAB2.5 hydrogels on the modulation of inflammatory response on day 7, the rats were sacrificed, and the tissues at the wound sites were collected for the analysis of the pro- and anti- inflammatory factors by RT-qPCR. It was found that both the blank HA and HA/SAB2.5 hydrogels could downregulate the expression of pro-inflammatory factor IL-1β and upregulate the expression of anti-inflammatory factor IL-10 on day 7, indicating their effects on the promotion of transition from pro-inflammatory phase towards anti-inflammatory/pro-healing phases in the wounds, with HA/SAB2.5 showing better capacities (Figure 5E,F). In the final stage of observation on day 14, the hydrogel-treated groups showed significantly faster healing rates than the control group, with normalized wound areas of (93.48 ± 1.74%) and (90.41 ± 2.61%) for HA/SAB2.5 and blank HA, in comparison with (85.58 ± 3.07%) for the control group.

**Figure 6:**
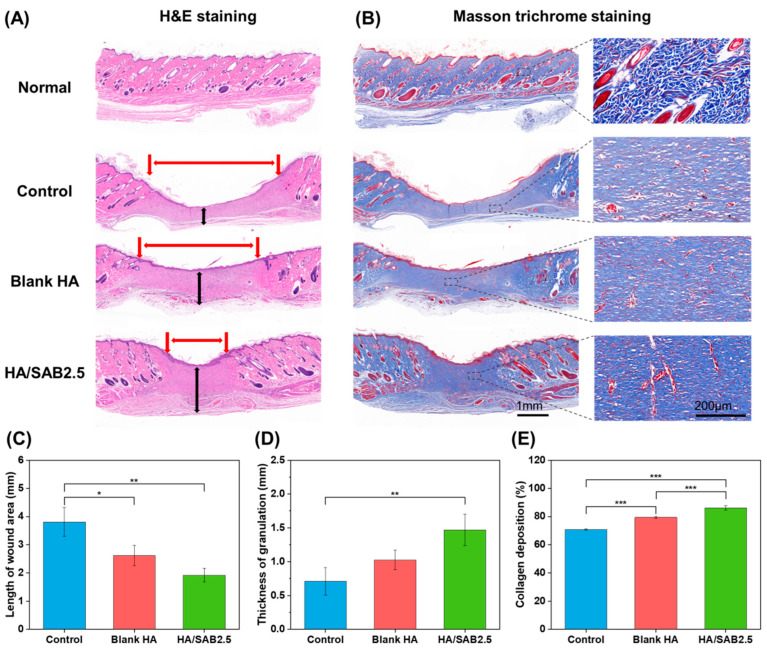
Histological evaluation of regenerated skin. (**A**) Representative images of H&E staining (**A**) (scale bar = 1 mm; red arrow: wound site length; black arrow: granulation tissue) and Masson trichrome staining (**B**) (scale bar = 1 mm/200 μm) for control, blank HA, and salvianolic acid B (SAB)-loaded HA hydrogel (HA/SAB2.5) on day 14. (**C**–**E**) Quantification of the wound site length, granulation tissue thickness, and collagen deposition of wound sites on day 14. Data represent mean ± SD, n = 3; * *p* < 0.05; ** *p* < 0.01; *** *p* < 0.001.

To further analyze the therapeutic effects at the histological level, H&E and Masson trichrome staining were conducted on the regenerated skin tissues collected on day 14. The H&E staining images are shown in Figure 6A, and the quantified data of the remaining dermal space length and thickness of granulation are presented in Figure 6C,D. The results showed that the remaining dermal space lengths of wounds in the control group were the longest (3.81 ± 0.51 mm), while they were shorter in the blank HA hydrogel group (2.62 ± 0.36 mm), and the HA/SAB2.5-treated group had the shortest remaining dermal space length (1.92 ± 0.4 mm). In addition, the regenerated granulation tissue of the blank HA group showed a medium thickness (1.02 ± 0.14 mm) and the HA/SAB2.5 group showed the highest thickness (1.47 ± 0.23 mm) (Figure 6A,D). The Masson trichrome staining results (Figure 6B,E) suggested a similar tendency compared to H&E staining, showing that the HA/SAB2.5 hydrogel-treated group had the most collagen deposition.

**Figure 7:**
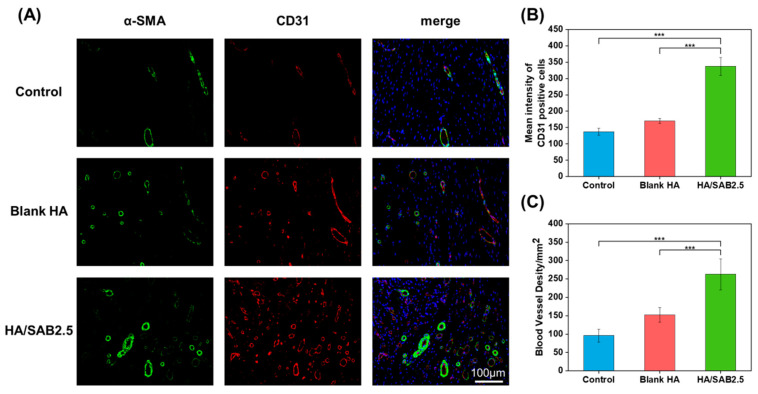
HA hydrogels promoted angiogenesis during wound healing process. (**A**) Immunofluorescence staining images of α-SMA (green), CD31 (red) and DAPI (blue) on day 14 post wounding for control, blank HA, and salvianolic acid B (SAB)-loaded HA hydrogel (HA/SAB2.5) (scale bar = 100 μm). (**B**) Mean intensity of CD31-positive cells in wounds. (**C**) Quantitative graph of blood vessel density on day 14 corresponding to α-SMA-positive staining in diabetic wounds. Data represent mean ± SD, n = 3; *** *p* < 0.001.

The angiogenesis-promoting abilities of the hydrogels was evaluated by immunofluorescence staining. As shown in Figure 7A, neovascularization occurred in all groups, characterized by the visualization of CD31-positive cells (red) surrounded by α-SMA-positive cells (green). The quantified data of the mean intensity of CD31-positive cells and quantified blood vessel density in wounds are shown in Figure 7B,C. The HA/SAB2.5 group exerted the highest expression of CD31 and α-SMA compared to the blank HA and control groups, demonstrating superior angiogenesis-promoting abilities.

## 3. Discussion

The wound healing cascade is generally divided into four coordinated phases: hemostasis, inflammation, proliferation, and remodeling [40]. Chronic diabetic wounds are mainly manifested in hyperglycemia, persistent inflammation, and insufficient angiogenesis [41]. A high glucose environment prevents the immune system from functioning effectively, causing the increased expression of pro-inflammatory cytokines [42]. Besides this, high glucose levels also trigger microvascular dysfunction, leading to ischemia and tissue necrosis, further resulting in delayed vascularization and impaired diabetic wound healing [43].

In this study, we fabricated a self-healing hydrogel via the covalent reaction of the hydrazide groups of HA-ADH and the aldehyde groups of OHA. According to the rheological assessment, the hydrogel returned to its initial modulus after three cycles of periodic strain changes, demonstrating the rapid and efficient self-healing properties of the HA hydrogel (Figure 2G). This result was also consistent with the previous reconstruction experiment of two pre-gelled hydrogels (Figure 1D(iii)). Favorable biocompatibility is a fundamental requirement for hydrogels in clinical applications. According to the cell proliferation results evaluated using a CCK-8 assay (Figure 3A), extracts of HA/SAB2.5 hydrogel had the best cell proliferation effect in contrast to blank HA and HA/SAB1 hydrogel. In addition, HA/SAB2.5 showed the best healing properties among all SAB-loaded hydrogel groups in a diabetic full-thickness skin defect model (Figure S1). Therefore, the HA/SAB2.5 hydrogel was selected for further exploration.

Salvianolic acid B (SAB) is one of the main active ingredients of *Salvia miltiorrhiza*, exhibiting excellent anti-inflammatory and pro-angiogenesis effects reported by others [44]. In this study, SAB was confirmed to reduce the mRNA expression of IL-1β and ccr7 (pro-inflammatory cytokine) and increase the mRNA expression of CD206 (M2 marker) in (LPS + IFN-γ)-stimulated or IL-4-stimulated THP-1-derived-macrophages in a high glucose environment (Figure 4A–C), demonstrating the function of SAB in the suppression of inflammation and the promotion of M2 polarization/pro-healing in diabetic wound environments.

Diabetic wound healing was significantly enhanced by the application of the HA/SAB2.5 hydrogel. As shown in Figure 5B–D, it was found that the HA/SAB2.5 hydrogel exhibited a faster wound contraction speed than other groups. In addition, wound healing with the HA/SAB2.5 hydrogel was accompanied by the acceleration of granulation growth, collagen deposition, and the improvement of angiogenesis (Figure 6A–E), indicating the multifunctional properties of the HA/SAB hydrogel during the proliferation and remodeling phases of wound healing. Due to the anti-inflammatory effects of SAB, the HA/SAB2.5 hydrogel reduced the mRNA expression of IL-1β and increased the mRNA expression of IL-10 significantly (Figure 5E,F). Moreover, the densities of CD31- and α-SMA-positive cells were significantly higher in the HA/SAB2.5 hydrogel-treated group in contrast to the control and blank HA groups (Figure 7A–C), indicating the superior angiogenesis-promoting abilities of the HA/SAB2.5 hydrogel. These results consistently confirmed that the HA/SAB hydrogel possesses great potential for the acceleration of diabetic wound healing by promoting anti-inflammatory and angiogenesis effects.

## 4. Materials and Methods

### 4.1. Materials, Cells and Animals

Sodium hyaluronate (HA, 200–400 kDa), sodium periodate (NaIO_4_), 1-(3-Dimethylaminopropyl)-3-ethylcarbodiimide hydrochloride (EDC), N-Hydroxy succinimide (NHS), and adipic dihydrazide (ADH) were obtained from Aladdin (Shanghai, China). RPMI1640 medium, Dulbecco’s Modified Eagle’s medium (DMEM, 4.5 g/L of glucose), and fetal bovine serum (FBS) were provided by Gibco (Sao Paulo, Brazil). Trypsin and antibiotics (50 units/mL penicillin and 50 units/mL streptomycin) were obtained from Cienry Biotechnology (Zhejiang, China). Salvianolic acid B (SAB) was purchased from Aifa Biotechnology (Chengdu, China).

The human acute monocytic leukemia cell line THP-1 was purchased from Meisen CTCC (Zhejiang, China) and was cultured in an RPMI1640 medium with 10% fetal bovine serum at 37 °C and 5% CO_2_. Mouse embryo fibroblast (NIH/3T3) was purchased from China Center for Type Culture Collection (CCTCC, Wuhan, China) and was cultured in DMEM medium with 10% fetal bovine serum at 37 °C and 5% CO_2_.

Male Sprague Dawley rats (180–200 g) were provided by Shanghai Sippe-Bk Lab Animal Co., Ltd., Shanghai, China. The rats were housed in SPF-level animal facilities at 25 °C, with free access to food and water. All the animal experiments were approved by Zhejiang Chinese Medical University Laboratory Animal Research Center (ZCMULARC).

### 4.2. Synthesis and Characterization of HA-ADH and OHA

HA-ADH was synthesized via the covalent cross-linking of aldehyde groups in oxidized HA (OHA) and hydrazide groups in adipic dihydrazide (ADH)-modified HA (HA-ADH) with the formation of reversible acylhydrazone bonds [45]. (Figure 1A). Briefly, HA (1 g, 2% *w*/*v*) was dispersed in 50 mL of MES buffer (pH value was around 5.4), and then, 7.5 mmol EDC and 7.5 mmol NHS were added and stirred for 1 h and 3 h, respectively, to activate the HA carboxylate groups. Afterwards, 5 mmol ADH was added and stirred for 24 h at room temperature. Finally, the reaction solution was dialyzed (MWCO = 3500) against NaCl (5 g/L) for 1 day and placed in deionized water for another 2 days, and then lyophilized. The product was characterized by ^1^H nuclear magnetic resonance (^1^H NMR) in D_2_O (Avanc III 600 MHz, BRUKER, Karlsruhe, German). OHA was synthesized by an oxidation reaction of HA using sodium periodate according to the previous method with some modifications [46]. (Figure 1A). Briefly, HA (1 g, 1% *w*/*v*) was dissolved in 100 mL of distilled water, and 5 mL of NaIO_4_ (0.5 M) was added dropwise to the HA solution; then, the reaction solution was stirred for 12 h in the dark at room temperature. Thereafter, excess amounts of ethylene glycol were added to terminate the oxidation process. Finally, the reaction mixture was dialyzed (MWCO = 3500) against deionized water for 3 days and then lyophilized. The product was characterized by Fourier transform infrared spectroscopy (FT-IR, VERTEX 70v, Bruker, German).

### 4.3. Morphology and Rheological Properties of the Hydrogels

The lyophilized HA-ADH and OHA were dissolved in PBS to form a 3% (*w*/*v*) solution. The HA hydrogels were prepared by mixing the HA-ADH and OHA at equal volume with a double-inlet syringe. The HA hydrogels with SAB concentrations of 0, 1, 2.5, and 5 mg/mL were prepared by loading the corresponding concentration of SAB into the precursors of HA hydrogel, and they were named blank HA, HA/SAB1, HA/SAB2.5, and HA/SAB5, respectively.

To obtain samples for the morphology test, the hydrogels were freshly prepared by mixing OHA and HA-ADH solution with the double-inlet syringe and injected into a 5 mL tube. After the hydrogels had completely gelled, the hydrogels in the tube were frozen quickly in liquid nitrogen and lyophilized for 48 h. Then, the lyophilized hydrogels were cut by cross-section, coated with a layer of gold film, and then captured by a field emission scanning electron microscope (SEM SU8010, Hitachi, Tokyo, Japan) with three images from each sample. Afterwards, 20 pores of each hydrogel were randomly selected for analysis of the mean pore size by ImageJ 2 software.

The rheology properties of the hydrogels were evaluated by a rheometer (MCR302, Anton Paar, Graz, Austria) with PP25 flat plates. The hydrogel precursors were injected using the double-inlet syringe and placed between parallel plates with a gap of 1 mm. Afterwards, the time sweep test was immediately performed at 25 °C at the constant frequency of 10 rad/s and 1% strain to investigate the gelation time of the hydrogel by comparing the values of storage modulus (G′) and loss modulus (G″). After the hydrogel was completely gelled according to the time sweep test curves (G′ and G″ achieve plateau), a strain sweep test was conducted with oscillatory strains from 0.1% to 1000% and a constant frequency of 10 rad/s. According to the results of the strain sweep, an angular frequency sweep from 0.1 to 10 rad/s at a constant strain of 1% strain was also performed. Furthermore, the self-healing property of the blank HA and HA/SAB2.5 hydrogels was evaluated by periodic strain changes of 1% and 300% every 200 s, with a frequency of 10 rad/s.

### 4.4. Degradation Performance and Drug Release Profile In Vitro

The degradation profiles of the hydrogels were determined gravimetrically. The known initial weight of hydrogels was immersed in PBS (pH = 7.4) at 37 °C. At different time points (6 h, 12 h, 24 h, 36 h, 48 h, 60 h, 72 h, 84 h, and 96 h), the samples were collected, gently blotted with filter paper, and then weighed. All experiments were carried out in triplicate. The degradation ratio of the hydrogels was calculated using the following formula: Degradation ratio (%) = (W_0_ − W_t_)/W_0_ × 100%, where W_0_ is the initial weight of the hydrogel and W_t_ is the weight of the hydrogel at interval time points of degradation.

The release of SAB from the HA/SAB hydrogels was determined in PBS. The HA/SAB hydrogels (2 mL) were immersed in 5 mL of PBS (pH = 7.4) at 37 °C. At predetermined time intervals (6 h, 12 h, 24 h, 36 h, 48 h, 60 h, 72 h, 84 h, and 96 h), 2 mL of the supernatant was collected and replaced with an equal volume of fresh PBS. All experiments were carried out in triplicate. After all samples were collected, the concentration of SAB in the supernatant was calculated according to a standard curve obtained at an absorbance wavelength of 286 nm in an ultraviolet/visible (UV/Vis) spectrophotometer (2800, Unico, Franksville, Wisconsin, USA). The following equation was used to calculate the percentage of drug release: Cumulative release of SAB (%) = (Released SAB/Total of SAB) × 100%.

### 4.5. Cytocompatibility Evaluation of the Hydrogels

The cytocompatibility of the hydrogels was determined with mouse embryo fibroblasts (NIH/3T3) with both a Cell Counting Kit-8 assay (CCK-8, Biosharp, Anhui, China) and LIVE/DEAD staining assay (Solarbio, Beijing, China). Hydrogels were immersed in PBS (pH = 7.4) for 24 h, and the extracts were filtered to degerm using a 0.22 μm microporous membrane (Biosharp, Anhui, China) for subsequent experiments.

For the CCK-8 assay, NIH/3T3 cells were seeded with an initial density of 1 × 10^4^ cells/well in a 24-well plate and incubated at 37 °C for 24 h in a 5% CO_2_ incubator. HA hydrogel extracts diluted to a concentration of 20% were added and cultured for another 24 and 72 h. Subsequently, the culture medium was removed and washed once with PBS, and then 100 μL of DMEM containing 10% (*v*/*v*) CCK-8 was added to each well and incubated for 2 h at 37 °C in the dark. The absorbance at 450 nm was read on a microplate reader (SpectraMax Plus 384, Molecular Devices, San Jose, CA, USA). Untreated cells served as the control group, and wells containing medium without cells served as the blank group.

For the LIVE/DEAD staining assay, the same protocol for cell seeding and culturing was used. After incubation with 20% hydrogel extracts for 24 and 72 h, the culture medium was removed and washed once with PBS. Following the manufacturer’s instructions, 100 μL of staining solution was added per well. After incubating for 15 min at 37 °C in the dark, the stained cells were visualized using fluorescence microscopy (Leica DMI4000 B, Wetzlar, German) with viable cells having been stained green and dead cells having been stained red.

### 4.6. Inflammatory Regulation of SAB In Vitro

The effects of SAB in inflammatory regulation were evaluated by RT-qPCR using lipopolysaccharide (LPS)-stimulated THP-1-derived macrophages in a high glucose environment.

Firstly, THP-1 cells were pre-treated with 100 ng/mL phorbol 12-myristate 13-acetate (PMA, Sigma, St. Louis, MI, USA) for 48 h to differentiate into macrophages prior to an additional 24 h of rest. Then, THP-1-derived macrophages were differentiated into M1 phenotypes after having been incubated with 100 ng/mL lipopolysaccharide (LPS, Sigma, USA) and 20 ng/mL Interferon-γ (IFN-γ, Solarbio, Beijing, China) for 24 h in a high glucose environment (25 mM). In addition, THP-1-derived macrophages were differentiated into M2 phenotypes after incubation with 20 ng/mL IL-4 for 48 h in a high glucose environment (25 mM). At the same time, 100 μM of SAB was added simultaneously to examine the effect of SAB in modulating inflammatory cytokine production.

For the RT-qPCR analysis, cells were gently washed once with PBS and lysed in 1 mL of Trizol reagent (Invitrogen, Renfrew, UK) on ice for 5 min; then, 200 μL chloroform was added to the collected total cell lysate, mixed, and left for 15 min. After centrifugation at 12,000 rpm for 15 min, the precipitate was discarded, and an equal volume of isopropanol was added to the supernatant for 20 min. The supernatant was then recentrifuged for 15 min, and the precipitate was washed twice with 75% ethanol and air dried before adding DEPC-treated water. Finally, pure RNA was obtained and quantified with a NanoDrop ONE Microvolume UV-Vis Spectrophotometer (Thermo Scientific, Waltham, MA, USA). cDNA was synthesized by a ReverTra Ace qPCR RT Kit (TOYOBO, Osaka, Japan), and RT-qPCR was conducted with SYBR^®^ Green Realtime PCR Master Mix (TOYOBO, Japan). The gene expressions of IL-1β, ccr7 and CD206 in THP-1-derived macrophages were analyzed by the 2^−ΔΔCt^ method. The primer sequences are listed in Table 1.

THP-1-derived macrophages were stimulated with 1 μg/mL LPS for 24 h in a high glucose environment (25 mM), and 200 μM of SAB was added simultaneously to investigate the potential anti-inflammatory mechanisms.

### 4.7. In Vivo Diabetic Wound Healing Assessment

For the diabetic wound healing assessment, male SD rats of 180–200 g were selected and fed adaptively for one week. After fasting for 16 h, streptozotocin (STZ, Yeasen Biotech, Shanghai, China), degermed by a 0.22 μm filter, was injected intraperitoneally at a dose of 65 mg/kg (freshly prepared from 0.1 M sodium citrate buffer, pH 4.2–4.5). Blood was collected from the tail vein every 7 days, and blood glucose was measured by a blood glucose meter. After 2 weeks, rats were considered to be a successful diabetic model when the blood glucose levels had exceeded 16.7 mmol/L.

All diabetic rats were subjected to surgery to establish the cutaneous full-thickness wounds. Briefly, diabetic rats were anaesthetized by the intraperitoneal injection of Zoletil 50 (40 mg/kg), then depilated and sterilized with 75% ethanol. The full-thickness skin defect model was established on the backs of rats with 10 mm diameter biopsy punches.

The rats were randomized into three groups: the control group, blank HA hydrogel group, and HA/SAB2.5 hydrogel group (nine rats in each group). The hydrogels were added to the wound site with a syringe and held in place with 3 M Tegaderm film. The control group was given the same amount of normal saline. Using a ruler, wounds were photographed on day 0, 3, 7, and 14, respectively. The wound area of each group was measured using Image J 2 software, and the wound healing rate was calculated according to the following formula: wound healing rate (%) = (A_0_ − A_t_)/A_0_ × 100%, where A_0_ and A_t_ represent the wound area of day 0 and test time, respectively.

The gene expressions of IL-1β and IL-10 in the wound area on day 7 were measured using RT-qPCR analysis. The wound samples were homogenized with Trizol reagent using a tissue grinder (LC-MY-20, LICHEN, Shanghai, China). The methods of RNA extraction and RT-qPCR were carried out as described above. The primers are listed in Table 2.

### 4.8. Histology and Immunofluorescence Staining

To assess granulation tissue thicknesses, remaining dermal space length, and collagen deposition on the wound sites, the regenerated skins were excised on day 14.

After being fixed with 4% *w*/*v* paraformaldehyde solution and embedded in molten paraffin, the tissues were cut into 3 μm thick sections for staining with H&E and Masson trichrome according to the specification, and then, images were collected with the scanner (Pannoramic MIDI, 3DHISTECH, Budapest, Hungary).

The immunofluorescence staining of CD31 and α-SMA (markers of endothelial cells and neovascularization) [34] was also used to observe neovascularization in the wound area. After dewaxing, hydration, and antigen retrieval, the slides were blocked with 5% BSA at room temperature for 30 min and incubated with CD31 rabbit polyclonal antibody (1:300, GB113151, Servicebio, Wuhan, China) and α-SMA mouse monoclonal antibody (1:200, GB13044, Servicebio, Wuhan, China) at 4 °C overnight. After washing with PBS, secondary antibodies Cy3-conjugated goat anti-rabbit IgG (1:300, Servicebio, Wuhan, China) and Alexa Flour 488-conjugated goat anti-mouse IgG (1:400, Servicebio, Wuhan, China) were added and incubated at 37 °C for 1 h. The slides were then mounted with an antifade mounting medium containing DAPI. Finally, the slides were visualized using a virtual slide microscope (VS120-S6-W, OLYMPUS, Tokyo, Japan) and quantitatively analyzed by Image J.

### 4.9. Statistical Analysis

The statistical analysis was carried out using GraphPad Prism 8.0 software. Data were presented as mean ± standard deviations (SDs). A one-way ANOVA with Tukey’s multiple comparison test was used to assess the difference between multiple groups. In all experiments, a *p* value less than 0.05 was considered statistically significant.

## 5. Conclusions

**Figure 8:**
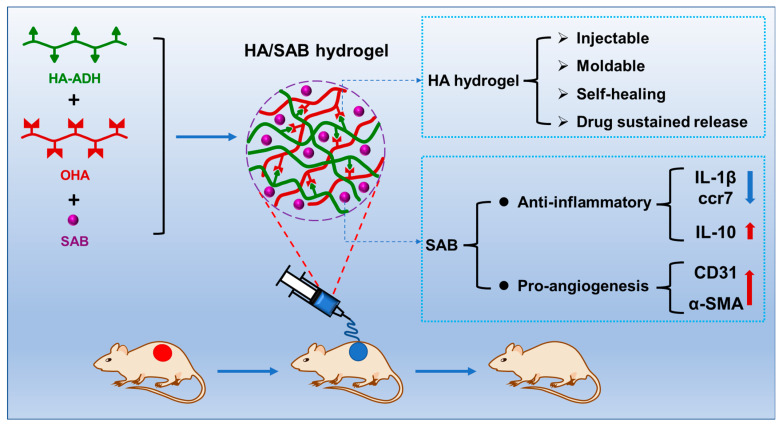
Salvianolic acid B-loaded HA self-healing hydrogel promotes diabetic wound healing through promotion of anti-inflammation and angiogenesis. Red arrow: HA/SAB hydrogel increased the expression of IL-10, CD31 and α-SMA. Blue arrow: HA/SAB hydrogel decreased the expression of IL-1β and ccr7. Red circular: wound sites established on the back of diabetic rats. Blue circular: wound sites treated with hydrogels.

In summary, we successfully developed a multifunctional hydrogel with good injectable and biodegradable properties, a sustainable release capacity of SAB, appropriate mechanical properties, and excellent self-healing properties. In vitro cell experiments showed that the HA/SAB hydrogels could promote cell proliferation and exhibit excellent cytocompatibility. In addition, SAB reduced the pro-inflammatory cytokine (IL-1β and ccr7) production in THP-1-derived macrophages in a high glucose environment, indicating the anti-inflammatory potential in a diabetic wound environment. Furthermore, wounds treated with the HA/SAB2.5 hydrogel showed a faster wound healing rate, reduced inflammatory response, shorter remaining dermal space length, thicker granulation tissue formation, more collagen deposition, and enhanced vascularization, in contrast to the blank HA hydrogel and control groups. Overall, our results demonstrate that the fabricated HA/SAB self-healing hydrogel is promising as a wound dressing to accelerate diabetic wound healing through the promotion of anti-inflammation and angiogenesis (Figure 8).

## Figures and Tables

**Table 1:** The sequences of cell RT-qPCR primers.

Primer Name	Primer Sequence (5′-3′)
Humo IL-1β	F: AATCTCACAGCAGCATCTCGACAAG
	R: TCCACGGGCAAGACATAGGTAGC
Humo ccr7	F: ATGGTGATCGGCTTTCTGGT
	R: CCAGGACCACCCCATTGTAG
Humo CD206	F: CCACAGTTATGCCTACCATGCC
	R: TCCCTCCAAAGCCTATACAAGC
Humo GAPDH	F: TGACATCAAGAAGGTGGTGAAGCAG
	R: GTGTCGCTGTTGAAGTCAGAGGAG

**Table 2:** The sequences of rat RT-qPCR primers.

Primer Name	Primer Sequence
Rat IL-1β	F: CTCAGAGCCATAAGAAAACCGT
	R: GACAATGCTGCCTCGTGACC
Rat IL-10	F: TATGTTGCCTGCTCTTACTGGCT
	R: GCAGTTATTGTCACCCCGGAT
Rat GAPDH	F: GACATGCCGCCTGGAGAAAC
	R: AGCCCAGGATGCCCTTTAGT

## Data Availability

The data that support the findings of this study are available on request from the corresponding author.

## Supplementary Materials

The following supporting information can be downloaded at: https://www.mdpi.com/article/10.3390/ijms24076844/s1.

## References

[1] Banday M.Z., Sameer A.S., Nissar S. (2020). Pathophysiology of diabetes: An overview. Avicenna J. Med..

[2] McDermott K., Fang M., Boulton A.J.M., Selvin E., Hicks C.W. (2023). Etiology, Epidemiology, and Disparities in the Burden of Diabetic Foot Ulcers. Diabetes Care.

[3] Rehak L., Giurato L., Meloni M., Panunzi A., Manti G.M., Uccioli L. (2022). The Immune-Centric Revolution in the Diabetic Foot: Monocytes and Lymphocytes Role in Wound Healing and Tissue Regeneration—A Narrative Review. J. Clin. Med..

[4] Kharaziha M., Baidya A., Annabi N. (2021). Rational Design of Immunomodulatory Hydrogels for Chronic Wound Healing. Adv. Mater..

[5] Nguyen T.T., Jones J.I., Wolter W.R., Perez R.L., Schroeder V.A., Champion M.M., Hesek D., Lee M., Suckow M.A., Mobashery S. (2020). Hyperbaric oxygen therapy accelerates wound healing in diabetic mice by decreasing active matrix metalloproteinase-9. Wound Repair Regen..

[6] Dunnill C., Patton T., Brennan J., Barrett J., Dryden M., Cooke J., Leaper D., Georgopoulos N.T. (2017). Reactive oxygen species (ROS) and wound healing: The functional role of ROS and emerging ROS-modulating technologies for augmentation of the healing process. Int. Wound J..

[7] Matoori S., Veves A., Mooney D.J. (2021). Advanced bandages for diabetic wound healing. Sci. Transl. Med..

[8] Esakkimuthukumar M., Swaroop A.K., Patnaik S.K., Kumar R.R., Praveen T.K., Naik M.R., Jubie S. (2022). A novel family of small molecule HIF-1 alpha stabilizers for the treatment of diabetic wounds; an integrated in silico, in vitro, and in vivo strategy. RSC Adv..

[9] Patel S., Srivastava S., Singh M.R., Singh D. (2019). Mechanistic insight into diabetic wounds: Pathogenesis, molecular targets and treatment strategies to pace wound healing. Biomed. Pharmacother..

[10] Lyttle B.D., Vaughn A.E., Bardill J.R., Apte A., Gallagher L.T., Zgheib C., Liechty K.W. (2023). Effects of microRNAs on angiogenesis in diabetic wounds. Front. Med..

[11] Asadi N., Pazoki-Toroudi H., Del Bakhshayesh A.R., Akbarzadeh A., Davaran S., Annabi N. (2021). Multifunctional hydrogels for wound healing: Special focus on biomacromolecular based hydrogels. Int. J. Biol. Macromol..

[12] Koehler J., Brandl F.P., Goepferich A.M. (2018). Hydrogel wound dressings for bioactive treatment of acute and chronic wounds. Eur. Polym. J..

[13] Zeng Q., Qian Y., Huang Y., Ding F., Qi X., Shen J. (2021). Polydopamine nanoparticle-dotted food gum hydrogel with excellent antibacterial activity and rapid shape adaptability for accelerated bacteria-infected wound healing. Bioact. Mater..

[14] Graça M.F.P., Miguel S.P., Cabral C.S.D., Correia I.J. (2020). Hyaluronic acid—Based wound dressings: A review. Carbohydr. Polym..

[15] Burdick J.A., Prestwich G.D. (2011). Hyaluronic acid hydrogels for biomedical applications. Adv. Mater..

[16] Kwon M.Y., Wang C., Galarraga J.H., Puré E., Han L., Burdick J.A. (2019). Influence of hyaluronic acid modification on CD44 binding towards the design of hydrogel biomaterials. Biomaterials.

[17] Gwon K., Kim E., Tae G. (2017). Heparin-hyaluronic acid hydrogel in support of cellular activities of 3D encapsulated adipose derived stem cells. Acta Biomater..

[18] Vu T.T., Gulfam M., Jo S.-H., Rizwan A., Joo S.-B., Lee B., Park S.-H., Lim K.T. (2023). The effect of molecular weight and chemical structure of cross-linkers on the properties of redox-responsive hyaluronic acid hydrogels. Int. J. Biol. Macromol..

[19] Yang B., Song J., Jiang Y., Li M., Wei J., Qin J., Peng W., Lasaosa F.L., He Y., Mao H. (2020). Injectable Adhesive Self-Healing Multicross-Linked Double-Network Hydrogel Facilitates Full-Thickness Skin Wound Healing. ACS Appl. Mater. Interfaces.

[20] Wang X., Xu P., Yao Z., Fang Q., Feng L., Guo R., Cheng B. (2019). Preparation of Antimicrobial Hyaluronic Acid/Quaternized Chitosan Hydrogels for the Promotion of Seawater-Immersion Wound Healing. Front. Bioeng. Biotechnol..

[21] Guan S., Li Y., Cheng C., Gao X., Gu X., Han X., Ye H. (2020). Manufacture of pH- and HAase-responsive hydrogels with on-demand and continuous antibacterial activity for full-thickness wound healing. Int. J. Biol. Macromol..

[22] Moura L.I., Dias A.M., Carvalho E., de Sousa H.C. (2013). Recent advances on the development of wound dressings for diabetic foot ulcer treatment—A review. Acta Biomater..

[23] Taylor D.L., Panhuis M.I.H. (2016). Self-Healing Hydrogels. Adv. Mater..

[24] Kim J.W., Kim S., Jeong Y.R., Kim J., Kim D.S., Keum K., Lee H., Ha J.S. (2022). Self-healing strain-responsive electrochromic display based on a multiple crosslinked network hydrogel. Chem. Eng. J..

[25] del Olmo J.A., Alonso J.M., Saez-Martinez V., Benito-Cid S., Moreno-Benitez I., Bengoa-Larrauri M., Perez-Gonzalez R., Vilas-Vilela J.L., Perez-Alvarez L. (2022). Self-healing, antibacterial and anti-inflammatory chitosan-PEG hydrogels for ulcerated skin wound healing and drug delivery. Biomater. Adv..

[26] Li S., Dong Q., Peng X., Chen Y., Yang H., Xu W., Zhao Y., Xiao P., Zhou Y. (2022). Self-Healing Hyaluronic Acid Nanocomposite Hydrogels with Platelet-Rich Plasma Impregnated for Skin Regeneration. ACS Nano.

[27] Yang R., Liu X., Ren Y., Xue W., Liu S., Wang P., Zhao M., Xu H., Chi B. (2021). Injectable adaptive self-healing hyaluronic acid/poly (γ-glutamic acid) hydrogel for cutaneous wound healing. Acta Biomater..

[28] Zhang M., Huang Y., Pan W., Tong X., Zeng Q., Su T., Qi X., Shen J. (2021). Polydopamine-incorporated dextran hydrogel drug carrier with tailorable structure for wound healing. Carbohydr. Polym..

[29] Choudhary M., Chhabra P., Tyagi A., Singh H. (2021). Scar free healing of full thickness diabetic wounds: A unique combination of silver nanoparticles as antimicrobial agent, calcium alginate nanoparticles as hemostatic agent, fresh blood as nutrient/growth factor supplier and chitosan as base matrix. Int. J. Biol. Macromol..

[30] Goh M., Hwang Y., Tae G. (2016). Epidermal growth factor loaded heparin-based hydrogel sheet for skin wound healing. Carbohydr. Polym..

[31] Rahim M.A., Kristufek S.L., Pan S., Richardson J.J., Caruso F. (2019). Phenolic Building Blocks for the Assembly of Functional Materials. Angew. Chem. Int. Ed..

[32] Qian Y., Zheng Y., Jin J., Wu X., Xu K., Dai M., Niu Q., Zheng H., He X., Shen J. (2022). Immunoregulation in Diabetic Wound Repair with a Photoenhanced Glycyrrhizic Acid Hydrogel Scaffold. Adv. Mater..

[33] Soares R.D.F., Campos M.G.N., Ribeiro G.P., Salles B.C.C., Cardoso N.S., Ribeiro J.R., Souza R.M., Leme K.C., Soares C.B., de Oliveira C.M. (2020). Development of a chitosan hydrogel containing flavonoids extracted from *Passiflora edulis* leaves and the evaluation of its antioxidant and wound healing properties for the treatment of skin lesions in diabetic mice. J. Biomed. Mater. Res. Part A.

[34] Yang H., Song L., Sun B., Chu D., Yang L., Li M., Li H., Dai Y., Yu Z., Guo J. (2021). Modulation of macrophages by a paeoniflorin-loaded hyaluronic acid-based hydrogel promotes diabetic wound healing. Mater. Today Bio.

[35] Lee H.G., Kwon S., Moon S.K., Cho S.Y., Park S.U., Jung W.S., Park J.M., Ko C.N., Cho K.H. (2023). Neuroprotective Effects of Geopung-Chunghyuldan Based on Its Salvianolic Acid B Content Using an in Vivo Stroke Model. Curr. Issues Mol. Biol..

[36] Liu H., Ma S., Xia H., Lou H., Zhu F., Sun L. (2018). Anti-inflammatory activities and potential mechanisms of phenolic acids isolated from *Salvia miltiorrhiza* f. alba roots in THP-1 macrophages. J. Ethnopharmacol..

[37] Xiao Z., Liu W., Mu Y.P., Zhang H., Wang X.N., Zhao C.Q., Chen J.M., Liu P. (2020). Pharmacological Effects of Salvianolic Acid B against Oxidative Damage. Front. Pharmacol..

[38] Li Y., Zhang X., Cui L., Chen R., Zhang Y., Zhang C., Zhu X., He T., Shen Z., Dong L. (2017). Salvianolic acids enhance cerebral angiogenesis and neurological recovery by activating JAK2/STAT3 signaling pathway after ischemic stroke in mice. J. Neurochem..

[39] Patil C., Patil M., Patil M. (2018). Studies on Synthesis of Aldimines: Part-I. Synthesis, Characterization and Biological Activity of Aldimines from Benzaldehyde with variedly substituted anilines. Recent Res. Sci. Technol..

[40] Kalantari K., Mostafavi E., Afifi A.M., Izadiyan Z., Jahangirian H., Rafiee-Moghaddam R., Webster T.J. (2020). Wound dressings functionalized with silver nanoparticles: Promises and pitfalls. Nanoscale.

[41] Davis F.M., Kimball A., Boniakowski A., Gallagher K. (2018). Dysfunctional Wound Healing in Diabetic Foot Ulcers: New Crossroads. Curr. Diabetes Rep..

[42] Scopelliti F., Cattani C., Dimartino V., Mirisola C., Cavani A. (2022). Platelet Derivatives and the Immunomodulation of Wound Healing. Int. J. Mol. Sci..

[43] Augustine R., Hasan A., Patan N.K., Dalvi Y.B., Varghese R., Antony A., Unni R.N., Sandhyarani N., Al Moustafa A.E. (2020). Cerium Oxide Nanoparticle Incorporated Electrospun Poly(3-hydroxybutyrate-co-3-hydroxyvalerate) Membranes for Diabetic Wound Healing Applications. Acs Biomater. Sci. Eng..

[44] Sun J.M., Ho C.K., Gao Y., Chong C.H., Zheng D.N., Zhang Y.F., Yu L. (2021). Salvianolic acid-B improves fat graft survival by promoting proliferation and adipogenesis. Stem Cell Res. Ther..

[45] Cai C., Zhang X., Li Y., Liu X., Wang S., Lu M., Yan X., Deng L., Liu S., Wang F. (2022). Self-Healing Hydrogel Embodied with Macrophage-Regulation and Responsive-Gene-Silencing Properties for Synergistic Prevention of Peritendinous Adhesion. Adv. Mater..

[46] Roh H.H., Kim H.S., Kim C., Lee K.Y. (2021). 3D Printing of Polysaccharide-Based Self-Healing Hydrogel Reinforced with Alginate for Secondary Cross-Linking. Biomedicines.

