# Supplementary material for: Salvianolic-Acid-B-Loaded HA Self-Healing Hydrogel Promotes Diabetic Wound Healing through Promotion of Anti-Inflammation and Angiogenesis
Source: Int J Mol Sci. 2023 Apr 6;24(7):6844. doi: 10.3390/ijms24076844 (PMC10095058; doi:10.3390/ijms24076844)
Supplement: Supplementary file 1 [file ijms-24-06844-s001.zip › ijms-2272103-supplementary.pdf]

Salvianolic acid B-loaded HA self-healing hydrogel promotes diabetic wound healing through promotion of anti-inflammation and angiogenesis

Guoying Zhou[1,§], Jiayan Zhu[1,§], Liang Jin[1], Jing Chen[1], Ruojiao Xu[1], Yali Zhao[1], Tingzi Yan[2,*], Haitong Wan[1,*]

[1]College of Life Science, Zhejiang Chinese Medical University, Hangzhou, 310053, China; gyzhou87@126.com (G.Z.)

[2]College of Material, Chemistry and Chemical Engineering, Hangzhou Normal University, Hangzhou, 311121, China

[§]These authors contributed equally to this work and should be considered as co-first authors

***Address correspondence to:**

Haitong Wan, Zhejiang Chinese Medical University, 548 Binwen Road, Hangzhou, 310053, China. E-mail address: whtong@126.com

Tingzi Yan, Hangzhou Normal University, 2318 Yu Hang Tang Road, Hangzhou, 311121, China. E-mail addresses: tzyan@hznu.edu.cn

In vivo diabetic wound healing assessment

All diabetic rats were subjected to surgery to establish the cutaneous full-thickness wounds. Briefly, diabetic rats were anaesthetized by intraperitoneal injection of Zoletil 50 (40 mg/kg), depilated and sterilized with 75% ethanol, the full-thickness skin defect model was established on the back of rats with 10 mm diameter biopsy punches.

The diabetic rats were then randomly divided into five groups: control group, blank HA hydrogel group, HA/SAB1 hydrogel group, HA/SAB2.5 hydrogel group and HA/SAB5 hydrogel group (6 rats in each group). The hydrogels were added to the wound site with a syringe and held in place with 3M Tegaderm film. The control group was given the same amount of normal saline. Using a ruler, wounds were photographed on days 0, 3, 7 and 14. The wound area of each group was measured using Image J software, and the wound healing rate was calculated according to the following formula: wound healing rate (%) = $(A_0 - A_t) / A_0 \times 100\%$, where A_0 and A_t represent the wound area of day 0 and test time, respectively.

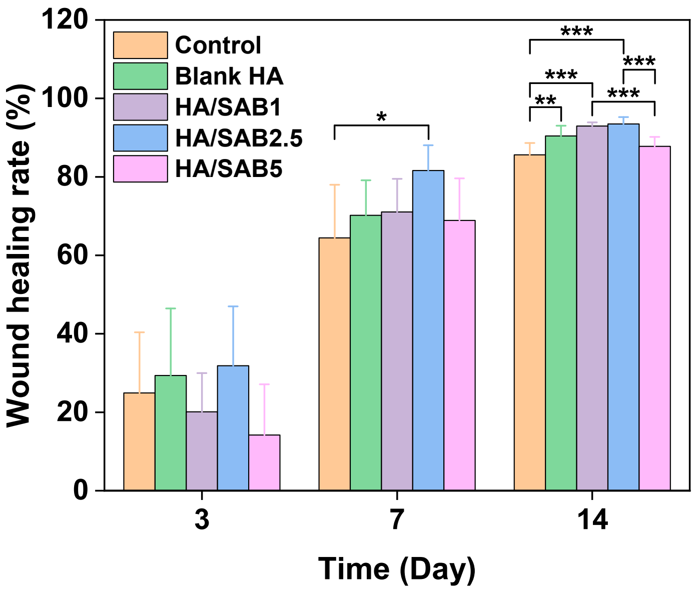

Figure S1. Quantitative data of wound healing rate on day 3, 7 and 14 following treatment of the blank HA, HA/SAB1, HA/SAB2.5, and HA/SAB5 hydrogels, n = 6. Data represent mean ± SD; * $p < 0.05$; ** $p < 0.01$; *** $p < 0.001$.

As shown in Figure S1, HA/SAB2.5 hydrogel-treated group displayed faster wound healing rate compared with the control group at day 7. Furthermore, all blank HA and HA/SAB-treated groups showed superior effects in accelerating wound healing by contrast to the control group at day 14. Thereupon, the HA/SAB1 and HA/SAB2.5 groups showed the best healing effects. Taken together with the results of cellular experiments, HA/SAB2.5 was selected among the HA/SAB samples for further histological and immunofluorescence studies.
